# A comparative study on the prevalence of musculoskeletal complaints among musicians and non-musicians

**DOI:** 10.1186/1471-2474-14-9

**Published:** 2013-01-04

**Authors:** Laura M Kok, Theodora PM Vliet Vlieland, Marta Fiocco, Rob GHH Nelissen

**Affiliations:** 1 Department of Orthopedics J11-R, Leiden University Medical Center, P.O. Box 9600, 2300, RC Leiden, the Netherlands

**Keywords:** Musculoskeletal diseases, Epidemiology, Musicians, Occupational diseases, Prevalence, Upper extremity, Cumulative trauma disorders

## Abstract

**Background:**

Research comparing the frequency of musculoskeletal complaints between musicians and non-musicians is scarce. The aim of this study was to compare the prevalence of musculoskeletal complaints between musicians and non-musicians.

**Methods:**

A cross-sectional study in 3215 students from three music academies (n = 345) and one medical school (n = 2870) in The Netherlands was performed, using an electronic questionnaire. The questionnaire included socio-demographic characteristics, use of music instruments and the occurrence of musculoskeletal complaints in six body regions. Questions were related to musculoskeletal complaints over the last twelve months and at the time of the questionnaire. Chi-square, t-tests and Kruskal-Wallis tests were used for comparison between the two groups. The association between musculoskeletal complaints and possible predictors was analyzed using a logistic and Poisson regression.

**Results:**

Eighty-seven music academy students and 503 medical students returned the questionnaire, of which respectively eighty-three and 494 were included in the study. Seventy-four music academy students (89.2%) reported one or more musculoskeletal complaints during the last twelve months, compared to 384 (77.9%) medical students (p = 0.019). Moreover 52 music academy students (62.7%) and 211 medical students (42.7%) reported current musculoskeletal complaints (p = 0.001). The Odds ratio (OR) for the development of musculoskeletal complaints during the last twelve months in music academy students versus medical students is 2.33 (95% CI 1.61–3.05, p = 0.022). The OR at the time of the questionnaire is 2.25 (95% CI 1.77–2.73, p = 0.001). The total number of complaints have been modeled by employing a Poisson regression; the results show that non-musicians have on average less complaints than musicians (p = 0.01). The adjusted means are 2.90 (95% CI 2.18–3.63) and 1.83 (95% CI 1.63–2.04) respectively for musicians and non-musicians. Regarding the localization of complaints, music academy students reported more complaints concerning the right hand, wrists, left elbow, shoulders, neck, jaw and mouth in contrast to medical students.

**Conclusions:**

Musculoskeletal complaints are significantly more common among musicians compared to non-musicians, mainly due to a higher number of upper extremity complaints.

## Background

Musculoskeletal complaints are a common problem in the general population. Nearly 75% of the Dutch population aged 25 years and older suffered from a complaint of the musculoskeletal system during a one-year period [1]. These complaints are a major cause of limitations in daily activities, health care usage and work disability [2-4].

Apart from musculoskeletal complaints leading to work disability, some occupations may cause specific work-related musculoskeletal complaints. It has been consistently demonstrated that jobs with frequently repeated movements like computer use and work with high physical demands are associated with musculoskeletal complaints [2,3,5]. Also psychosocial work characteristics and increased stress symptoms such as high job demands and lack of control or social support are related with musculoskeletal complaints [6].

Musicians have a work environment with high musculoskeletal and psychosocial demands [7]. In order to play their instrument, musicians need to frequently repeat physically strenuous movements. On average a musician plays 1300 hours a year in an ergonomically unfavorable position [8]. Instruments, requiring different positions and playing techniques, are associated with a different prevalence of musculoskeletal complaints [9-14]. Musculoskeletal complaints have been reported frequently [14-16], and they have a considerable physical but also psychological, social and financial impact on musicians.

Previous research shows a prevalence of musculoskeletal complaints varying from 39% up to 90% in adult musicians [13-17]. The severity of the complaints studied and the relation with playing the instrument (‘playing related musculoskeletal disorders’ [18]) have a considerable impact on the prevalence. It is difficult to interpret these results since musculoskeletal complaints are also common in the general population. Actually, there are only two small studies comparing the prevalence of musculoskeletal complaints between musicians and non-musicians, with contrasting outcomes [19,20]. Fry et al. [20] compared 98 secondary school students playing in the school orchestra to an age- and sex matched group of students who did not play. Occurrence of playing-related pain was 63% in girls and 49% in boys. A questionnaire concerning playing-related pain in the instrument-playing group was only compared with hand pain in the control group, without further specifications in localization of the playing-related pain. Roach et al. [19] examined 99 instrumentalists, and 159 non-instrumentalist university students. The former did not report more joint pain than the latter, but showed more pain in the upper-body than in the lower. A methodological flaw in that study was that the two groups were not comparable for age and sex, nor was corrected for this difference.

Given the scarcity of data and research on musculoskeletal complaints in musicians, this study aimed to compare prevalence, localization and associations between type of instrument and musculoskeletal complaints between musicians and non-musicians.

## Methods

### Study design and patients

This cross-sectional study compared year- and point prevalence of musculoskeletal complaints among music students and medical students. The study was performed at four Dutch institutions: the Royal Conservatoire, The Hague; the CODARTS University for the Arts, Rotterdam; the Amsterdam School of the Arts; and the medical faculty of the Leiden University between February and May 2011. All Dutch-speaking students of the above mentioned music academies with a classical instrument as main subject (singers and conductors were excluded) and medical students from the Leiden University (all of them speaking Dutch) received an invitation. They were selected from the student registries of the four centers. All eligible students received an e- mail with an invitation to complete the online questionnaire, with a reminder invitation three weeks after the first. After completing the questionnaire, students younger than 18 or older than 30 years were excluded. The Medical Ethical Committee (CME) of the Leiden University Medical Center approved the protocol. Informed consent was obtained from all participants.

### Assessments

The electronic questionnaire included the following items.

#### Sociodemographic characteristics and general health

Age, gender, length, weight, right/left-handed, study-year (bachelor 1 till 4, master 1 or 2), playing an instrument and study (music academy student / medical student playing an instrument / medical student not playing an instrument), main instrument (violin, viola, cello, base, piano/keyboard, guitar/ mandolin, bassoon, oboe, clarinet, flute / piccolo, horn, trombone, tuba, harp, percussion, recorder and other, in which the participants had to fill in their instrument) were asked. The instruments were divided in five categories: (1) bowed strings, (2) plucked strings, (3) woodwinds, (4) brass and (5) percussion and keyboards. For students playing an instrument, information like the number of years already spent to play the instrument and the average number of hours per week devoted to practice was asked. In addition, the questionnaire included questions concerning smoking (none / up to a half package a day / half to one package a day / more than one package a day), alcohol (number of glasses per week), and sports (number of hours per week).

#### Musculoskeletal complaints

Since no validated scores were available for musicians, a questionnaire on musculoskeletal complaints was constructed. The first author, who is both Medical Doctor and has a Master degree in music, extensively discussed the questionnaire with colleagues in the medical and performing arts field. The score consisted of 144 questions on the occurrence of complaints in six specific body regions, subdivided in 21 localizations (yes/no). Questions on each of these regions started by asking about complaints of *-the specific body region*- during the last 12 months’, The first body region ‘elbows, wrists and hands’ was subdivided in six localizations (elbow, wrist and hand left and right). The second one ‘neck shoulders and upper back’ was subdivided in four localizations (shoulders, neck, upper back). The third region ‘lower back’ was not subdivided. The fourth one ‘hips and knees’ was subdivided in four localizations (hip and knee left and right). ‘Ankles and feet’ (fifth region) was subdivided in four sub regions (ankle and foot left and right). The last region ‘jaw and mouth’ was subdivided in the two regions. The total prevalence score was calculated by adding all subjects with at least one complaint. The prevalence in a specific body region was also calculated by adding all subjects with at least one complaint in that particular body region. If the above mentioned question concerning complaints during the last twelve months was positive, it was also asked whether the complaint was still present and at which localization of the body (yes/no). The same procedure was applied to each body region of interest.

The total number of students with complaints was calculated by adding all students with at least one complaint. One-year prevalence was calculated by dividing the percent of subjects with complaints during the last twelve months by one hundred. The point-prevalence was calculated by dividing the percentage of subjects reporting at least one complaint which was present at the time of the questionnaire by one hundred.

### Statistical analysis

All Statistical analysis were performed in SPSS version 18. For continuous normally distributed variables mean and standard deviation were calculated or median, in case of departure from the normal distribution the range have been computed. Comparisons between the two groups were performed by employing Chi-square, t-tests and Kruskal-Wallis tests. Complaints and the total number of complaints in the two groups have been investigated respectively by a univariate logistic and a Poisson regression. Details are given in the section results.

## Results

The questionnaire was sent to 345 musical and 2870 medical students. Initially, 590 students completed the questionnaire, 87 music academy students and 503 medical students, leading to response rates of 25.5% for the music academy students and 17.6% for the medical students (18.4% overall response rate). Thirty-three of the 135 students of the Royal Conservatory completed the questionnaire (response 24.4%), 26 of the 124 students of the Amsterdam school of the Arts (response 20.9%) and 24 of the 86 students of the CODARTS University for the arts (response 27.9%). Three subjects from the music academy group were excluded since they were younger than eighteen while eight subjects were excluded from the medical students group because they were older than 30 years. An additional two subjects were excluded because they were singers. Finally 577 students were included: 83 from the music academies and 494 from the medical school. In Table 1 the characteristics of the responders are illustrated.

**Table 1:** Baseline characteristics of music academy and medical students participating in a survey on musculoskeletal complaints

	**Music academy students (n = 83)**	**Medical students (n = 494)**	**Difference (p)**
Age (years) (mean (SD))	21.5 (2.2)	22.1 (2.6)	P = 0.062 ~
Gender (%)	Male: 22 (26.2%) Female: 62 (73.8%)	Male: 120 (24.3%) Female: 374 (75.7%)	P = 0.843 *
Study (%)	Bachelor: 72 (86.7%) Master: 11 (13.3%)	Bachelor: 248 Master: 246	P < 0.001 *
Smoking (%)	10 (11.9%)	26 (5.3%)	P = 0.019 *
Sport (hours in one week) (mean (SD))	2.2 (2.4)	3.0 (2.8)	P = 0.005 ~
Alcohol consumption (E/week) (mean (SD))	3.9 (4.5)	5.5 (6.9)	P = 0.090 ~
Body mass index (kg/m^2^) (mean (SD))	21.2 (3.0)	22.0 (2.5)	P = 0.001 ~
Hours of practicing the main musical instrument in one week (mean (SD))	20.7 (8.7)		
Experience (number of years playing the main musical instrument) (mean (SD))	13.0 (3.3)		
Hand preference (%)	Right: 71 (85.5%) Left: 12 (14.5%)	Right: 433 (87.7%) Left: 61 (12.3%)	P = 0.593 *

~ = Kruskal Wallis Test.

* = Chi-squared Test.

In the group of the medical students, 162 (32.8%) played an instrument. The instruments played by the music academy students were very different from the instruments played by the medical students; 29 (34.9%) music academy students played a bowed string instrument, 3 (3.6%) a plucked instrument, 27 (35.2%) a woodwind, 7 (8.4%) brass and 17 (20.5%) percussion or keyboard. Medical students played more often percussion or keyboard (73, 45.1%), or a plucked string instrument (39, 24.1%). Sixteen of them (9.9%) played a bowed string instrument, 26 (16.0%) played a woodwind and 8 (9.4%) played brass.

The music academy students were comparable with the medical students with respect to age, gender, length, alcohol consumption and hand preference. However, they differed with respect to the degree of the study (bachelor/master), hours of sport in a week, smoking, and body mass index (music academy students are lighter than medical students) (Table 1).

Seventy-four music academy students (89.2%) reported one or more musculoskeletal complaints during the last twelve months, compared to 384 (77.9%) medical students (p = 0.019, Table 2). Among music academy students 52 (62.7%) reported current musculoskeletal complaints, while in the medical group 211 (42.7%, p = 0.001). The OR for point prevalence for musculoskeletal complaints in the music academy students was 2.25 (95% CI 1.77–2.73, p = 0.001), for year prevalence 2.33 (95% CI 1.61–3.05, p = 0.022). The total number of complaints (the number of localizations in one subject) have been modeled by employing a Poisson regression; the results show that non-musicians had on average less complaints than musicians (p = 0.01). The adjusted means were 2.90 (95% CI 2.18–3.63) and 1.83 (95% CI 1.63–2.04) respectively for musicians and non-musicians.

**Table 2:** Musculoskeletal complaints among music academy and medical students specified by body region

		**Music academy students (n = 83)**	**Medical students (n = 494)**	**Difference (p)**
Elbows, wrists, hands (%)	Subjects with complaints during the last twelve months	40 (48.2%)	109 (22%)	p < 0.001 *
	Subjects with complaints at the time of filling in the questionnaire	14 (16.9%)	39 (8%)	p = 0.009 *
	Reported number of complaints of the elbows, wrists and hands (0–6) (Mean (SD))	0.7 (0.98)	0.27 (0.556)	P < 0.001 ~
Neck, shoulders, upper back (%)	Subjects with complaints during the last twelve months	65 (78.3%)	233 (47%)	P < 0.001 *
	Subjects with complaints at the time of filling in the questionnaire	39 (47.0%)	96 (19%)	p < 0.001 *
	Reported number of complaints of the neck, shoulders and upper back (0–4) (Mean (SD))	1.2 (1.00)	0.56 (0.664)	P < 0.001 ~
Lower back (%)	Subjects with complaints during the last twelve months	33 (39.8%)	191 (39%)	P = 0.860 *
	Subjects with complaints at the time of filling in the questionnaire	19 (22.9%)	63 (13%)	p = 0.014 *
Hips, knees (%)	Subjects with complaints during the last twelve months	11 (13.3%)	146 (30%%)	P = 0.002 *
	Subjects with complaints at the time of filling in the questionnaire	6 (7.2%)	71 (14%)	p = 0.077 *
	Reported number of complaints of the hips and knees (0–4) (Mean (SD))	0.2 (0.57)	0.34 (0.569)	P = 0.017 ~
Ankles, feet (%)	Subjects with complaints during the last twelve months	7 (8.4%)	82 (17%)	P = 0.057 *
	Subjects with complaints at the time of filling in the questionnaire	6 (7.2%)	41 (8%)	p = 0.741 *
	Reported number of complaints of the ankles and feet (0–4) (Mean (SD))	0.1 (0.57)	0.19 (0.470)	P = 0.201 ~
Jaw, mouth (%)	Subjects with complaints during the last twelve months	21 (25.3%)	38 (8%)	P <0.001 *
	Subjects with complaints at the time of filling in the questionnaire	9 (10.8%)	24 (5%)	p = 0.030 *
	Reported number of complaints of the jaw and mouth (0–2) (Mean (SD))	0.3 (0.50)	0.08 (0.297)	P = 0.001 ~
**Total (%)**	**Subjects with complaints during the last twelve months**	**74 (89.2%)**	**384 (78%)**	P = 0.019 *
	**Subjects with complaints at the time of filling in the questionnaire**	**52 (62.7%)**	**211 (43%)**	P = 0.001 *
	**Reported total number of complaints (0–21) (Mean (SD))**	**2.9 (2.61)**	**1.83 (1.516)**	**P < 0.001 ~**

~ = Kruskal Wallis Test.

* = Chi-squared Test.

More music academy students reported complaints during the last twelve months on the body regions elbows, wrists and hands, the neck, shoulders and upper back and the jaw and mouth compared to medical students (Table 2). Contrary, music academy students reported fewer complaints of hips and knees. The proportions of students reporting complaints of the lower back or ankles and feet were similar between the two groups. In Table 3 complaints during the last twelve months specified by exact localizations are presented, showing differences between right and left sides.

**Table 3:** Musculoskeletal complaints during the last twelve months among music academy and medical students specified by localization

		**Music academy students (n = 83)**	**Medical students (n = 494)**	**Difference (p)**
Hand	Right	14 (16.9%)	35 (7.1%)	P = 0.003*
	Left	7 (8.4%)	21 (4.3%)	P = 0.101*
Wrist	Right	14 (16.9%)	31 (6.3%)	P = 0.001*
	Left	13 (15.7%)	27 (5.5%)	P = 0.001*
Elbow	Right	2 (2.4%)	9 (1.8%)	P = 0.717*
	Left	6 (7.2%)	8 (1.6%)	P = 0.002*
Shoulder	Right	25 (30.1%)	42 (8.5%)	P < 0.001*
	Left	23 (27.7%)	32 (6.5%)	P < 0.001*
Neck		38 (45.8%)	135 (27.3%)	P = 0.001*
Upper back		16 (19.3%)	68 (13.8%)	P = 0.188*
Lower back		33 (39.8%)	191 (39%)	P = 0.860*
Knee	Right	5 (6.0%)	74 (15.0%)	P = 0.028*
	Left	5 (6.0%)	61 (12.3%)	P = 0.094*
Hip	Right	2 (2.4%)	13 (2.6%)	P = 0.906*
	Left	3 (3.6%)	22 (4.5%)	P = 0.728*
Ankle	Right	2 (2.4%)	29 (5.9%)	P = 0.196*
	Left	3 (3.6%)	32 (6.5%)	P = 0.312*
Foot	Right	5 (6.0%)	19 (3.8%)	P = 0.358*
	Left	2 (2.4%)	15 (3.0%)	P = 0.755*
Jaw		13 (15.7%)	31 (6.3%)	P = 0.003*
Mouth		9 (10.8%)	10 (2.0%)	P < 0.001*

* = Chi-squared Test.

With respect to the number of complaints (number of involved localizations/joints) reported, music academy students did report a higher number of complaints of elbows, wrists and hands (mean 0.67 (95% CI 0.46–0.88) versus 0.27 (95% CI 0.23–0.319), p < 0.001), shoulders, neck and upper back (1.24 (95% CI 1.03–1.45) versus 0.56 (95% CI 0.50–0.62)), p < 0.001) and on the jaw and mouth (0.27 (95% CI 0.17–0.37) versus 0.08 (95% CI 0.06–0.11), p = 0.001). No statistical significant differences in the number of complaints on the hips, knees, ankles, feet and lower back have been found.

Within medical students playing and not playing an instrument there were no significant differences except for a significant difference in the baseline factors BMI (p = 0.04) and study year (p = 0.025) and the number of facial complaints is different (p = 0.025). For all other outcomes there were no significant differences.

In Table 4 the occurrence of musculoskeletal complaints is compared between different instrument groups. The prevalence of musculoskeletal complaints was the highest in musicians who used a plucked string or percussion or a keyboard instrument. They were followed by the woodwind bowed string and brass players, but the differences between these groups of music academy students are not significant.

**Table 4:** Musculoskeletal complaints during the last twelve months in music academy students according to instrumental sections

	**Strings, bowed (n = 29)**	**Strings, plucked (n = 3)**	**Woodwinds (n = 27)**	**Brass (n = 7)**	**Percussion and keyboards (n = 17)**	**Difference (p)**
Musculoskeletal complaints during the last twelve months (year prevalence) (%)	24 (83%)	3 (100%)	25 (93%)	6 (86%)	16 (94%)	p =0.655
Musculoskeletal complaints at the moment of filling in the questionnaire (point prevalence) (%)	18 (62%)	3 (100%)	17 (63%)	2 (29%)	12 (71%)	p =0.221

The CODARTS University of the Arts had the highest number of students with musculoskeletal complaints (year prevalence of 95.8% and point prevalence of 66.7%). However, no significant differences between the three music academies have been found in this study.

## Discussion

Music academy students reported more musculoskeletal complaints compared to medical students. Shoulders, neck and upper back were the regions being most affected within the musician group, followed by hands and wrists. Differences in occurrence existed between the right and left side. Current complaints and complaints during the last year showed comparable results regarding the localization of the complaints.

Since playing an instrument will usually affect the upper extremity and the neck region, it is conceivable that musicians have more upper body-part complaints. However, medical students do report significant more lower-body-part complaints, others found the same distribution of musculoskeletal complaints [19]. A hypothesis is that music academy students possibly avoid sports which could easily invoke an injury to the upper extremity, which will have a direct impact on their instrument performance and thus career opportunities.

Musculoskeletal complaints are reported with different prevalence rates between instrument groups [8,10,21,22]. This study shows clear differences although the sample size in this study is too small to investigate associations between a specific instrument type and the occurrence of complaints. The type of instrument played is a known risk factor for the development of musculoskeletal complaints among musicians [9,10,13-15,22,8]. The difference in prevalence between instrument groups (strings, woodwinds, brass, keyboard, percussion) implies that mechanical overuse is an important factor, which is contrary to repetitive strain injuries in which psychosocial are predominant factors in the etiology and not the mechanical repetition as such [6,23].

Besides two small studies [19,20] with conflicting outcomes, no study comparing musicians with non-musicians with respect to musculoskeletal complaints have been performed before. Literature comparing the results of a musculoskeletal questionnaire among musicians with a general workforce sample does exist, however due to heterogeneity between study populations (e.g. age, sex, activities), different research questions and methodologies, no comparisons can be made [8].

Compared to other studies on professional and adolescent musicians [8-10,14,16,22], this research shows a relative high prevalence of complaints of the musculoskeletal system. A possible explanation could be related to questions formulated in the questionnaire. In many studies, pain is the only complaint questioned, while in this study also other musculoskeletal complaints are taken into account as well. The reason for our different approach is the fact that not all musculoskeletal problems are associated with pain, but nevertheless they can cause severe disability. Although pain is often one of the main complaints, sometimes other discomfort symptoms are the main problem, for example in focal dystonia (in which losing coordination without pain is the main complaint) [24,25]. Besides, we choose not to make a distinction between playing-and non-playing related musculoskeletal complaints as of course playing-related complaints do not exist in non-musicians. Most studies concerning the prevalence of musculoskeletal complaints, or ‘playing related musculoskeletal disorders’, in orchestras and music schools, show high prevalence rates [14-16], but in those studies no control group was used. Comparing the complaints between musicians and non-musicians is important since the prevalence of musculoskeletal complaints in the general population is high. Thus, the additional effect of the exposure to play a musical instrument by a musician cannot be evaluated, if an age and sex matched control group is absent.

Some limitations are present in our study. Compared to other studies using a mailed questionnaire [26] the response rate is low. A possible reason for the low response rate is the fact that the invitation for the questionnaire was sent by e-mail only twice. It was not possible to send a reminder in another form or perform a telephone interview. Possible selection bias due to the response rate should be kept in mind. By choosing medical students as a control group a possible selection bias might be present since these students might be more aware of health problems, and therefore they might report problems easier. On the other hand they might also consider musculoskeletal complaints as being of none importance or even ignoring them. This implies that the effect of this potential bias is unclear.

## Conclusions

This research emphasizes that musicians do have significant more musculoskeletal complaints than non-musicians, which seems to be associated with the part of the body which is used to play the instruments, (i.e. the upper body and upper extremity). Both medical doctors and teachers in music academies should be aware of this problem and an analysis of how the instruments are played is important to identify musculoskeletal complaints and might be important to start preventive measurements. Since the prevalence is high compared to the general population, research into effective interventions to prevent and treat musculoskeletal complaints among musicians is necessary.

## Competing interests

The authors declare that they have no competing interests.

## Authors’ contributions

LMK, TPMVV and RGHHN developed the design for this study. LMK collected the data. Statistical analysis was performed by LMK and MF. LMK was the main writer of the manuscript. MF, RGHHN and TPMVV have been involved in revising it critically. All authors read and approved the final manuscript.

## Pre-publication history

The pre-publication history for this paper can be accessed here:

http://www.biomedcentral.com/1471-2474/14/9/prepub
